# The role of maternal and child healthcare providers in identifying and supporting perinatal mental health disorders

**DOI:** 10.1371/journal.pone.0306265

**Published:** 2024-07-11

**Authors:** Carmen Kiraly, Betty Boyle-Duke, Liat Shklarski

**Affiliations:** 1 Harriet Rothkopf Heilbrunn School of Nursing, Long Island University, Brooklyn Campus, Brooklyn, New York, United States of America; 2 Primary Care Health Services, Barnard College, New York, New York, United States of America; 3 Social Work Program, School of Social Science and Human Services, Ramapo College, Mahwah, New Jersey, United States of America; Group for Technical Assistance / Asian College for Advance Studies, Purbanchal University, NEPAL

## Abstract

**Background:**

Perinatal depression (PND) is underdiagnosed in the clinical setting. This study explores the role of obstetricians, and other primary care providers of maternal and child healthcare in detecting, screening, and referring women during the perinatal period identified as depressed, anxious, or exhibiting other symptoms of mental health disorders.

**Method:**

Information was gathered from obstetricians (*n* = 16), and other primary care providers (pediatricians, nurse practitioners, physician assistants) (*n* = 85), on identifying and supporting childbearing women with symptoms of perinatal depression using an online survey.

**Results:**

Statistical comparisons across participant groups were adjusted for years of practice in the profession. Statistically significant differences were noted. Obstetricians inquired more about the mother’s social support network (*p* = .011) and addressed mothers that appeared sad, upset, or unhappy (*p* = .044) compared to other primary care providers. Other primary care providers were more likely to refer patients to mental health support services (*p* = .005), provide PND-related information in their waiting rooms (*p* = .008), and use the Edinburgh Postnatal Depression Scale (EPDS) (*p* = .027). There was also a significant difference in positively identifying eight symptoms of PND between provider groups. Obstetricians had higher rates of identifying the following symptoms: excessive crying (*p* < .001), feeling little or no attachment to the infant (*p* < .001), little feeling of enjoyment (*p* = .021), feelings of failure (*p* < .001), hopelessness (*p* < .001), agitation with self and infant (*p* < .001), fear of being alone with the infant (p = .011), and fear that these symptoms would last (*p* < .001).

**Conclusion:**

Although certain screening practices were performed well, especially by the obstetrician group, screening deficits were noted within each group, and screening practices differed between groups. Training offered to maternal child health primary care providers on addressing perinatal mental health disorders may help improve provider screening practices and detection of PND symptoms in perinatal women. PND screening that combines face-to-face open-ended interviews with standardized screening tools can enhance patient-provider communication, potentially improving PND detection rates and follow-up care in perinatal women.

## Introduction

Perinatal depression (PND) is a serious mental health concern that affects one in eight new mothers. Rates of postpartum depression (PPD) are higher among women who report depressive symptoms prior to or during their pregnancy [1]. Due to the stigma that is often associated with behavioral health diagnoses, this condition can easily go undetected and is often underdiagnosed [2]. Pregnancy and the period after delivery can be a stressful time for mothers as new or additional responsibilities emerge, hormonal as well as physical changes occur, and family dynamics are permanently altered. For some women, these changes can be overwhelming and trigger depressive symptoms, sadness, and even suicidal ideations. Postpartum depression (PPD) often manifests one or two months after delivery and is most prevalent within 12 months of the birth of the infant [1, 3], leaving obstetricians, and other maternal and child health primary care providers such as pediatricians, nurse practitioners and physician assistants, well-positioned to evaluate new mothers during the postpartum period and thereafter. Frequent and ongoing screening by primary care providers of perinatal healthcare is essential in helping to identify this condition as early as the antenatal period to ensure proper management of PPD since this condition not only affects the mother but also the newborn, depending on the mother’s symptoms or related behaviors [4]. Depression during the antenatal and postpartum periods can include persistent sadness, difficulty concentrating, loss of interest in previously enjoyed activities, feelings of hopelessness, guilt, and worthlessness and may also be accompanied by anxiety [5, 6].

Deficits in the delivery of perinatal mental healthcare exist partly because screenings during antenatal and postpartum follow-up visits are not always performed. According to data reported in the Pregnancy Risk Assessment Monitoring System (PRAMS) survey in 2018, based on patients’ self-reports, one in eight women in the postpartum period and one in five women in the antenatal period were not asked about depression during visits, suggesting an inadequate rate of provider screening with a higher prevalence of provider screening during postpartum compared to antenatal visits [1]. Similarly, other studies describe less than optimal rates of provider PND screening [7, 8], or when screening did occur, the purpose of the screening tool and/or screening results were not disclosed, nor was follow-up provided, causing mothers to perceive the screening as ineffective [9, 10]. Although screening for depression and other mental health symptoms is recommended during the entire perinatal period [11], data from studies [7–10], reflect overall lower than expected rates and/or inadequate PND screening. Moreover, screening methods used to detect AND and PPD within various clinical settings often lack standardization, and symptoms associated with depression are often discounted as normal changes associated with pregnancy and the period following the delivery of the infant [12].

Several studies reveal specific barriers to screening of mental health disorders in diverse populations of women in the perinatal period. Among these include mothers’ lack of knowledge about depression screening tools, reasons why the screening is being done, signs/symptoms of perinatal depression, cultural expectations related to motherhood, and perceived stigma associated with revealing mental health disorders to providers they do not know [7, 9, 13].

Other barriers include mothers’ perceptions that providers may lack sensitivity in addressing mental health issues or treat symptoms as expected hormonal changes [7], treat them with racial bias, or report them to Child Protective Services once they fill out the screening tool [9]. Given these factors, it is important to explore the role of obstetricians and other providers of maternal and child healthcare in detecting, screening, and referring perinatal women identified as being depressed, anxious, or exhibiting symptoms of mental health disorders. Additionally, enhancing patient-provider conversations about PND and assessing social support networks along with other factors that contribute to PND may mitigate patient disconnections with providers and thereby improve perinatal mental health screening overall [10].

The primary aim of this exploratory study was to gather information from obstetricians, and other primary care providers, specifically pediatricians, nurse practitioners and physician assistants, about provider practices in identifying, referring, and supporting women of childbearing age with symptoms of perinatal depression, anxiety, and other mental health disorders using an online screening survey. A second aim was to assess provider differences in perinatal screening practices in their patient populations. The authors hypothesized that there would be differences between obstetricians, and other primary care providers in the screening practices of perinatal depression and anxiety in their patients.

## Materials and methods

### Data collection procedure

In this paper, symptoms of depression occurring in the antenatal period will be referred to as *antenatal depression* (AND) and those occurring in the postpartum period will be termed ‘*PPD*.’ Additionally, since the term *perinatal* refers to the antenatal and postpartum periods up to and including the infant’s 12-month well-baby visit, the term *perinatal* will be used interchangeably with the terms *antenatal* and *postpartum*.

This research project received Institutional Review Board approval (Protocol #19/09-098) with exempt status. The study employed a quantitative approach, using a convenience sampling method. Participants were invited to partake in an online survey, distributed between October 1^st^, 2019, and February 18^th^, 2020. Information about the study was disseminated to obstetricians and other primary care providers via the researchers’ professional contacts (phone calls/emails), and Facebook. Online electronic consent was obtained from interested parties indicating that participation was voluntary and could be terminated at any time. Participants indicated their written consent by proceeding with the online survey at the beginning of the survey through an individual link that could only be submitted once. To maximize response rates, a follow-up invitation was sent to those who had not yet completed the survey two weeks after the initial invitation. Participants received a $5 electronic Starbucks gift card sent to their email address upon survey completion. Completion time for the survey was 15–20 minutes. Upon submission, the responses were automatically encrypted and transmitted to a secure database, ensuring the privacy and protection of participant data.

### Participant demographics

All participants were required to be over the age of 21, reside in the United States, and be providers of care to women of childbearing age at risk of experiencing AND or PPD. These criteria were established to ensure a level of professional experience and contextual relevance to the healthcare system in the United States. Practice locations within the US were not specified in the survey.

### Survey instrument and data management

Two online surveys were created by the researchers to generate data for the study using Qualtrics Core XM software (2019–2020) (Qualtrics, Provo, Utah), an online platform endorsed by the institution for the distribution of survey-based research. The Qualtrics platform uses individual links using email distribution, thus allowing only one opportunity for submission, and preventing multiple submissions of the surveys. Although *N* = 114 respondents agreed to the electronic consent to participate in the survey, 13 did not respond to any further questions; consequently, the effective sample size is *N* = 101 (16 obstetricians and 85 other providers). In survey 1, *The Role of the Obstetrician in Identifying and Supporting Women’s Mental Health Symptoms*, obstetricians (*n* = 16) were recruited for participation, and in survey 2, *The Role of Other Primary Care Providers in Identifying and Supporting Women’s Mental Health Symptoms*, pediatricians, nurse practitioners, and physician assistants whose scope of practice included the perinatal period were recruited (*n* = 85).

Content for the online electronic survey included six demographic questions that noted the participant’s gender, age, ethnicity, highest degree completed, years in the field, and employment status. Thirty questions pertaining to aspects of screening for mental health disorders by physicians and other healthcare providers in antenatal and postpartum women included level of comfort during the screening process, ability to recognize signs and symptoms of mental health disorders, screening tools used to guide the assessment, if scores were reviewed with the patient, if treatment options were offered, if follow-up was done, and if a referral to a psychiatrist and/or therapist was initiated, among others. Several of these questions required the use of one of two Likert response styles: *always*, *most of the time*, *about half the time*, *sometimes*, and *never*; or *strongly agree*, *agree*, *somewhat agree*, *neither agree nor disagree*, *somewhat disagree*, and *disagree*. Finally, participants were asked to identify among 19 symptoms, those which they believed were associated with AND and/or PPD in their patients. These symptoms included excessive crying, feeling numb, feeling little or no attachment to the infant, having no interest in things that use to give pleasure, feeling little enjoyment, no energy, feelings of failure, feeling overwhelmed by the smallest of tasks, hopelessness, agitation with self/others/infant, insomnia, anxiety, excessive sleeping, difficulty concentrating, changes in appetite, fear of being alone with the infant, intrusive thoughts of harming the infant or harmful things happening to the infant, difficulty shaking unwanted feelings, and fear the symptoms would last.

### Data analysis

Demographic characteristics and survey responses were summarized using descriptive statistics (counts and percentages). Demographic characteristics were compared across participant groups (obstetricians, other providers) using an exact version of Pearson’s Chi-squared test [14].

Survey responses related to AND, PPD, and mental health screening practices were compared across participant groups using the Cochran-Mantel-Haenszel (CMH) test, adjusting for potential imbalance in a key factor: years of practice in the profession. The same CMH approach was used to analyze symptoms considered by the participant to be related to AND, PPD, and anxiety. As this study was exploratory in nature, no adjustment for multiple comparisons was planned or implemented. No imputations of missing data were used; only observed data were analyzed. Statistical tests were 2-sided and p-values < 0.05 were considered statistically significant. All analyses were conducted using R version 4.3.1 [15].

## Results

### Sample characteristics

**Table 1** lists the demographic characteristics of the whole group (*N* = 101) and compares obstetricians (*n* = 16) to other primary care providers (*n* = 85). Most of the participants in each group were female (93.8% and 88.0%, respectively). Significant differences were found regarding age (*p* = 0.002) and race/ethnicity of the providers (*p* < 0.001). Obstetricians in the sample were more likely to be older (45–54 years of age) (56.2%) and Black or African American (43.8%) compared to other primary care providers who were more likely to be younger (25–34 years of age) (61.9%) and White (78.6%). Significant differences regarding level of education (*p* < 0.001) and how long providers had been practicing (*p* = 0.001) were noted with obstetricians having a higher level of education (75%) and being in practice for over 20 years (26.7%). Finally, significant differences were evident in the ages (*p* < 0.001), racial/ethnic groups (*p* < 0.001), and religious affiliations (*p* = 0.027) of the providers’ patient populations. Obstetricians were more likely to see patients that were between the ages of 15-to-35 years of age (66.7%), more racially/ethnically diverse (73.3%), and religiously diverse (64.3%).

**Table 1:** Demographic characteristics of participants. Total sample = 101^1^.

Variable	Obstetricians (n = 16)		Primary Care Providers (n = 85)		*p* value
	Number	%	Number	%	
**Gender**					0.684
Female	15	93.8	73	88.0	
Male	1	6.2	10	12.0	
**Age**					0.002*
25–34	2	12.5	52	61.9	
35–44	4	25.0	10	11.9	
45–54	9	56.2	16	19.0	
55–64	1	6.2	6	7.1	
**Ethnicity**					<0.001*
Asian	1	6.2	7	8.3	
Black or African American	7	43.8	3	3.6	
Hispanic or Latino	3	18.8	4	4.8	
Native Hawaiian or Pacific Islander	0	0.0	4	4.8	
White	3	18.8	66	78.6	
Other	2	12.5	0	0.0	
**Highest Degree**					<0.001*
Bachelor’s degree	0	0.0	6	7.1	
Professional degree	2	12.5	56	66.7	
Master’s degree	2	12.5	13	15.5	
Doctorate degree	12	75.0	9	10.7	
**Employment Status**					0.366
Employed for wages	12	80.0	52	64.2	
Self-employed	3	20.0	29	35.8	
**How long practicing**					0.001*
1–5 years	3	20.0	49	60.5	
6–10 years	3	20.0	11	13.6	
11–20 years	5	33.3	19	23.5	
Over 20 years	4	26.7	2	2.5	
**Population age of patients**					<0.001*
15–20	1	6.7	0	0.0	
15–25	1	6.7	0	0.0	
15–30	0	0.0	2	2.6	
15–35	10	66.7	1	1.3	
20–25	1	6.7	1	1.3	
20–35	2	13.3	0	0.0	
25–30	0	0.0	51	65.4	
25–35	0	0.0	21	26.9	
30–35	0	0.0	1	1.3	
35+	0	0.0	1	1.3	
**Population ethnicities**					<0.001*
Hispanic					
Black					
Caucasian					
Hispanic, Black					
Hispanic, Black, Caucasian					
Hispanic, Black, Caucasian, Asian					
**Population religions**					0.027*
Jewish	0	0.0	6	37.5	
Chistian	2	14.3	3	18.8	
Muslim	1	7.1	0	0.0	
Jewish, Christian	0	0.0	2	12.5	
Christian, Muslim	1	7.1	1	6.2	
Jewish, Christian, Muslim	1	7.1	0	0.0	
Jewish, Christian, Muslim, Hindu	9	64.3	4	25.0	

Column totals for Obstetricians and Other Providers will be less than 16 and 85, respectively, when survey question was not answered by all participants in the group.

### Provider perinatal screening practices

**Table 2** represents data for participants’ responses to questions related to AND, PPD, and anxiety/mental health symptom screening practices; statistical comparisons across participant groups were adjusted for years of practice in the profession. Statistically significant differences between the two groups of providers were noted with 100% obstetricians addressing mothers that looked “sad, tired, upset, unhappy, or with a flat affect” (*p* = 0.044) and 93.3% asking mothers about “social support and forms of help” (*p* = 0.011) *most of the time/alway*s compared to 66.2% and 51.9% of other primary care providers, respectively. Regarding offering mothers’ treatment options such as referral to a psychiatrist or therapist (*p* = 0.005), having PND brochures in the waiting room (*p* = 0.008), and using the Edinburgh Postnatal Depression Scale (EPDS) [16] (*p* = 0.027), significant differences were also noted between the groups. For these variables, other primary care providers referred patients to a psychiatrist (35.8%) or therapist (59.3%), provided patients with information on AND and PPD and anxiety in their waiting rooms (77.5%), and used the EPDS (72.8%) more than the obstetrician group.

**Table 2:** Perinatal screening practices: Participant responses to questions related to AND, PPD, and anxiety/mental health symptoms. Total sample = 101^1^.

Variables	Obstetricians (n = 16)		Primary Care Providers (n = 85)		CMH *p* value 2
	Number	%	Number	%	
**Use screening tool to identify PPD /anxiety**					0.081
Never	4	26.7	5	6.2	
Sometimes/About half the time	0	0.0	19	23.5	
Most of the time/Always	11	73.3	57	70.4	
**Review scores with patients.**					0.400
Never	3	20.0	3	3.8	
Sometimes/About half the time	4	26.7	24	30.0	
Most of the time/Always	8	53.3	53	66.2	
**Treatment options offered to patients** **who report mental health symptoms.**					0.005*
Other	4	26.7	1	1.2	
Recommend finding mental health provider	3	20.0	3	3.7	
Referral to psychiatrist	3	20.0	29	35.8	
Referral to therapist	5	33.3	48	59.3	
**Follow-up with mothers about mental/** **emotional health.**					0.648
Never	0	0.0	2	2.5	
Sometimes/About half the time	4	26.7	30	37.0	
Most of the time/Always	11	73.3	49	60.5	
**Ask about mental/emotional health in first visit.**					0.453
Never	1	6.7	0	0.0	
Sometimes/About half the time	5	33.3	23	28.4	
Most of the time/Always	9	60.0	58	71.6	
**Ask about mothers’ connection with infant during office visit.**					0.122
Sometimes/About half the time	2	13.3	33	40.7	
Most of the time/Always	13	86.7	48	59.3	
**Ask about mothers’ social support/forms of help**					0.011*
Sometimes/About half the time	1	6.7	39	48.1	
Most of the time/Always	14	93.3	42	51.9	
**Variables**	**Obstetricians (n = 16)**		**Other Primary Care Providers (n = 85)**		**CMH *p* value**
	**Number**	**%**	**Number**	**%**	
**Provide patients with information about pre/postpartum depression/anxiety**					0.376
Never	3	20.0	2	2.5	
Sometimes/About half the time	3	20.0	14	17.3	
Most of the time/Always	9	60.0	65	80.2	
**In the office waiting room there are brochures about pre/postpartum depression**					0.008*
Never	4	28.6	1	1.2	
Sometimes/About half the time	4	28.6	17	21.2	
Most of the time/Always	6	42.9	62	77.5	
**Do you use the Edinburgh Postnatal depression scale?**					0.027*
Never	5	33.3	5	6.2	
Sometimes/About half the time	0	0.0	17	21.0	
Most of the time/Always	10	66.7	59	72.8	
**Where do you see patients?**					0.065
A clinic	3	20.0	35	43.2	
A hospital	3	20.0	34	42.0	
Your office	8	53.3	11	13.6	
Other	1	6.7	1	1.2	
**Do you screen for depression/anxiety/mood disorders of the mother?**					0.125
Sometimes/About half the time	1	6.7	31	38.8	
Most of the time/Always	14	93.3	49	61.2	
**If a mother does not report mental health symptoms, do you still check/ask about their mental health?**					0.963
Never	1	6.7	1	1.2	
Sometimes/About half the time	2	13.3	26	32.1	
Most of the time/Always	12	80.0	54	66.7	
**Variables**	**Obstetricians (n = 16)**		**Other Primary Care Providers (n = 85)**		**CMH *p* value**
	**Number**	**%**	**Number**	**%**	
**If you notice the mother looking sad, tired, upset, unhappy, or with flat affect, do you address it with her?**					0.044*
Sometimes/About half the time	0	0.0	27	33.8	
Most of the time/Always	15	100	53	66.2	
**I usually do not have time to ask mother about mental health symptoms**					0.882
Strongly disagree/ disagree/somewhat disagree	8	53.3	27	33.3	
Neither agree nor disagree	2	13.3	9	11.1	
Somewhat agree/agree/strongly agree	5	33.3	45	55.6	
**The well-being of the mother is critical for the healthy development of an infant/child**					0.527
Strongly disagree/ disagree/somewhat disagree	0	0.0	6	7.4	
Neither agree nor disagree	0	0.0	2	2.5	
Somewhat agree/agree/strongly agree	15	100.0	73	90.1	
**A clinic visit is an opportunity to identify mothers who are experiencing mental health challenges.**					0.527
Strongly disagree/ disagree/somewhat disagree	0	0.0	4	4.9	
Neither agree nor disagree	0	0.0	4	4.9	
Somewhat agree/agree/strongly agree	15	100.0	73	90.1	
**I am not sure how to address perinatal mental health**					0.103
Strongly disagree/ disagree/somewhat disagree	11	73.3	31	38.3	
Neither agree nor disagree	2	13.3	10	12.3	
Somewhat agree/agree/strongly agree	2	13.3	40	49.4	

Column totals for Obstetricians and Other Providers will be less than 16 and 85, respectively, when survey question was not answered by all participants in the group.

2. *P*-value from Cochran-Mantel-Haenszel test, adjusting for years of practice in the profession.

The following describes results for survey questions that were not statistically significant after adjusting for years of practice, but nonetheless show a tendency for provider differences in perinatal screening practices in their patient populations. For example, for the question, “Do you screen for depression/anxiety/mood disorders of the mother,” 93.3% of obstetricians answered *most of the time/always* compared to 61% of other primary care providers, and 73% of obstetricians and 70.4% of other providers responded *most of the time/always* to using a screening tool to identify AND and/or PPD. Seventy-three percent of obstetricians and 60.5% of other providers followed up with mothers about mental/emotional health *most of the time/always*. Most obstetricians (86.7%) compared to 59.3% of other providers asked about the mother’s connection with her infant during the office visit *most of the time/always*, and there was a greater tendency for obstetricians to see patients in their office (53.3%) compared to other providers who were more likely to see patients in a hospital (42%) or clinic setting (43.2%). About half (49.4%) of other providers compared to 13.3% of obstetricians responded *somewhat agree/agree/strongly agree* to the question, “Not sure how to address perinatal mental health,” although almost all providers in both groups offered treatment referral options.

Finally, of the 11 participants in each group that answered, “What tool do you use?” two providers used the Patient Health Questionnaire-9 (PHQ-9) [17] and nine used the EPDS in the obstetrician group, and ten used the PHQ-9 and one used the EPDS in the other provider group. Thus, among those who use a screening tool, there was a greater tendency for other primary care providers to use the PHQ-9.

### Identification of mental health symptoms

**Table 3** describes survey responses from both groups as participants were asked to identify symptoms [18] they believed were associated with AND, PPD or anxiety in their patients; statistical comparisons across participant groups were adjusted for years of practice in the profession. Significant differences were noted between groups with regard to positively identifying the following symptoms: excessive crying (*p* < .001) (100% obstetricians), feeling little or no attachment to the infant (*p* < .001) (100% obstetricians), little feeling of enjoyment (*p* = .021) (80% obstetricians), feelings of failure (*p* < .001) (93.3% obstetricians), hopelessness (*p* < .001) (66.7% obstetricians), agitation with self and infant (*p* < .001) (86.7% obstetricians), fear of being alone with the infant (p = .011), (73.3% obstetricians), and fear that these symptoms would last (*p* < .001) (80% obstetricians). A symptom that approached significance in detecting differences between groups was anxiety (*p* = .054), with most obstetricians (86.7%) identifying it as a symptom associated with AND and/or PPD compared to 43.2% of other primary care providers.

**Table 3:** Identification of mental health symptoms. Total sample = 101^1^.

Variable	Obstetrician (n = 16)		Primary Care Providers (n = 85)		CMH *p* value ^2^
	Number	%	Number	%	
**Excessive crying**					< 0.001*
No	0	0.0	53	65.4	
Yes	15	100.0	28	34.6	
**Feeling numb**					0.658
No	6	40.0	28	34.6	
Yes	9	60.0	53	65.4	
**Feeling little to no attachment to the infant**					< 0.001*
No	0	0.0	55	67.9	
Yes	15	100.0	26	32.1	
**No interest in things that used to give pleasure**					0.992
No	4	26.7	31	38.3	
Yes	11	73.3	50	61.7	
**Little feeling of enjoyment**					0.021*
No	3	20.0	47	58.0	
Yes	12	80.0	34	42.0	
**No energy**					0.646
No	3	20.0	35	43.2	
Yes	12	80.0	46	56.8	
**Feelings of failure**					< 0.001*
No	1	6.7	49	60.5	
Yes	14	93.3	32	39.5	
**Feeling overwhelmed by the smallest tasks**					0.219
No	4	26.7	50	61.7	
Yes	11	73.3	31	38.3	
**Hopelessness**					< 0.001*
No	5	33.3	66	81.5	
Yes	10	66.7	15	18.5	
**Agitation with self, others, infant**					< 0.001*
No	2	13.3	69	85.2	
Yes	13	86.7	12	14.8	
**Insomnia**					0.113
No	3	20.0	45	55.6	
Yes	12	80.0	36	44.4	
**Anxiety**					0.054
No	2	13.3	46	56.8	
Yes	13	86.7	35	43.2	
**Excessive sleeping**					0.157
No	7	46.7	55	67.9	
Yes	8	53.3	26	32.1	
**Difficulty concentrating**					0.403
No	4	26.7	48	59.3	
Yes	11	73.3	33	40.7	
**Changes in appetite**					0.574
No	5	33.3	52	64.2	
Yes	10	66.7	29	35.8	
**Fear of being alone with the infant**					0.011*
No	4	26.7	61	75.3	
Yes	11	73.3	20	24.7	
**Intrusive thoughts of harming the infant or harmful things happening to the infant**					0.579
No	4	26.7	49	60.5	
Yes	11	73.3	32	39.5	
**Difficulty shaking unwanted feelings**					0.234
No	6	40.0	62	76.5	
Yes	9	60.0	19	23.5	
**Fear these symptoms will last**					< 0.001*
No	3	20.0	77	95.1	
Yes	12	80.0	4	4.9	

1. Column totals for Obstetricians and Other Providers will be less than 16 and 85, respectively, when the survey question was not

answered by all participants in the group.

2. P-value from Cochran-Mantel-Haenszel test, adjusting for years of practice in the profession.

## Discussion

Our study explored the role of obstetricians and other primary care providers (pediatricians, nurse practitioners, physician assistants) in screening for AND and PPD during clinical visits. The findings noted between provider groups support our hypothesis that there would be differences in mental health screening practices when caring for perinatal women. Statistically significant differences in screening practices between provider groups included referring women for treatment, use of the EPDS, having PND brochures in the providers’ waiting rooms, addressing maternal affect, asking about social support, and recognizing PND symptoms. Additionally, survey responses indicated numerous inadequacies within both provider groups (e.g., reviewing scores with patients, using a standardized screening tool, and following up with mothers about their emotional/mental health). Other studies support our findings and reflect overall inadequate rates of healthcare provider inquiry about PND: 79% [1], 56% [19], 67% [7], 66.3% [8], and 64.8% [20].

### Other modalities to assess for PND

Several studies [7, 9, 10, 13, 21] describe mothers’ preferences for engaging in face-to-face dialogues about PND with their provider, and believed the PND screening process would be more effective if providers reviewed the purpose of the screening tools, explained test scores, and provided timely follow-up referrals. Inquiring about a mother’s mood and her social environment is a valuable screening approach as it provides information on her emotional state and can alert the provider to the risk for PND [22], exceeding the benefits of a checklist alone [23]. Considering the variability in mental health screening practices among perinatal healthcare providers, in addition to using a standardized tool, a comprehensive screening interview that asks about mothers’ social support, connection to the infant, and provides a follow-up after a referral, may improve detection of mental health disorders in perinatal women [9, 24].

### Best practice recommendations for provider PND screening

The American Academy of Pediatrics (AAP) and the American College of Obstetricians and Gynecologists (ACOG) both recommend frequent mental health screenings at certain intervals during the perinatal period [25, 26]. The AAP has recommended screening at the 1-, 2-, 4-, and 6-month well-baby visits [25]. Moreover, screening up to the 12-month period following delivery is prudent since symptoms of depression, anxiety, and mental health disorders are commonly detected during this time. A longitudinal study on rates of PPD screening in mothers during well-child visits up to 12 months of the infant’s life found a 15% rate of first-time positive screens in the 6–12-month period, and a 23% rate of mothers screening positive at the 12-month well-baby visit [27]. Yet, screening inconsistencies are common. [28] compared primary care providers’ (pediatricians/nurse practitioners) screening practices during well-child visits: 29% screened according to AAP screening guidelines, 64% screened at least once during the 12-months of the infant’s life according to the National Association of Pediatric Nurse Practitioners (NAPNAP) [29] screening guidelines, and 31% did not meet either AAP or NAPNAP screening guidelines. [30] noted improved PPD screening, from 83% to 88%, by pediatric and family nurse practitioners, when the EPDS was implemented at 1-, 2-, and 6-month well-child visits. Another study using a 20-item survey examined pediatrician attitudes and knowledge with PPD screening [8]. Sixty-six percent (66.3%) stated recognizing signs of PPD in a mother, and of these, only 13% referred the at-risk mothers to a mental health professional. Lack of time (84%) and not being familiar with mental health resources (53.5) were reported as screening barriers. Pediatricians that received training on PPD had improved confidence with assessment (*p* = .002) and knowledge of PPD (*p* < .001) and were more likely to screen for PPD (*p* = .012) in their patients.

Although ACOG [26] has recommended screening for symptoms of depression at the first obstetric visit, at 24–28 weeks of pregnancy, and at the comprehensive 4-week postpartum assessment using a validated tool, PND screening by obstetric healthcare providers in the U.S. remain inconsistent. Screening rates across a large hospital system found that 65.1% of women were screened for depression during pregnancy, and 64.4% were screened in the postpartum period [20]. [19] found screening practices for PND inconsistent and lacking a standardized screening tool in a U.S. community women’s health clinic serving lower income minority women. After implementing an educational in-service on PND screening and electronic health record (EHR) documentation, rates of PND documentation in the patients’ EHR rose from 56% to 92.7% (p < .05). The project’s success was attributed to the educational in-service received by the staff and the addition of a patient checklist in the EHR to properly document EPDS scores [19]. [12] examined the feasibility of universal mandatory PPD screening with the EPDS tool by adding a “hard stop” feature to the electronic health records of a large cohort (2,102) of high-risk women returning for the 6-week post-partum follow-up visit. Results indicated that PPD screening was completed in 99.5% of the women.

### Training for providers

The uncertainty in addressing perinatal mental health issues and the variance in symptom recognition as seen in our study and in similar studies may be due to the providers’ lack of knowledge about perinatal mental health symptoms and comfort level with addressing symptoms [8]. Thus, all healthcare providers encountering perinatal women would benefit from in-service training on identifying symptoms associated with PND and addressing perinatal health issues to be better prepared to screen for this condition [8, 31]. Improving physicians and other healthcare providers’ knowledge of PND screening and the available resources for women with positive screens would undoubtedly remove some of the barriers that impede PND detection in the clinical setting and may prove beneficial to maternal mental health outcomes [8, 24, 32]. Our study’s findings, in conjunction with the findings of similar studies, underscore the need to improve provider training about PND as well as utilize a screening tool in maternal and child health primary care to improve rates in detection, referrals to mental health services, and follow-up care.

### Limitations

This exploratory study had several limitations. First, the participant sample was small, with more ‘other primary care providers’ than obstetricians, resulting in findings that may limit generalizability to other maternal and child healthcare providers in the U.S. Second, our surveys did not include questions regarding providers’ awareness of the significant risk factors associated with AND and PPD or if the providers screened for these risk factors during their patient contacts. Third, although the surveys were disseminated through the professional networks of the researchers who are located primarily in the northeastern United States, information on actual geographical locations (i.e., city or county) specifying where the providers treated their patients was not collected in the demographic data, thereby limiting generalizability to specific areas in the U.S.

## Conclusion

This research examined the role of obstetricians and other primary care providers in screening for PND and other mental health symptoms and assessed differences in screening practices between provider groups. Statistically significant differences were found in the screening practices between provider groups, including referring women for treatment, use of the EPDS tool, having PND brochures in the providers’ waiting rooms, addressing maternal affect, asking about social support, and recognizing PND symptoms. Additionally, although several of the providers’ PND screening practices were performed well (*mostly always/always*), especially by the obstetrician group (e.g., addressing maternal affect, asking about social support, recognizing PND symptoms), survey responses indicated screening deficits within both provider groups (e.g., reviewing scores with patients, using a standardized screening tool, following up with mothers about their emotional/mental health). Findings from other studies highlight similar suboptimal perinatal mental health screening rates. Future research should focus on enhancing training for perinatal healthcare providers to increase comfort with addressing mental health disorders in perinatal women and identifying symptoms associated with these conditions. Screening methods that incorporate face-to-face conversations about a mother’s social support network and connection to the infant, in conjunction with using a standardized screening tool, may be more effective at capturing the needs of diverse populations of perinatal women than with a screening tool alone. Our findings support the study’s research hypothesis and suggest a need for further research and potential policy changes to improve mental health care during the perinatal period.

## Supporting information

S1 File (DOCX)

S1 Data (XLSX)

## References

[1] Bauman B, Ko J, Cox S, D’Angelo D, Warner L, Folger S, et al. *Vital signs*: Postpartum depressive symptoms and provider discussions about perinatal depression—United States, 2018. MMWR Morbidity and Mortality Weekly Report. 2020. 69:575–581. 10.15585/mmwr.mm6919a2externalicon. PMC7238954 32407302

[2] Leboffe E, Pietragallo H, Doug L, Goudong L, Djibril B, Chuang C. Impact of the 2015 ACOG screening guidelines on prevalence of postpartum depression among privately insured women. *Obstetrics & Gynecology*, 2020. 135, 118S. 10.1016/j.jad.2023.02.020 36764363

[3] Roehr B. American Psychiatric Association explains DSM-5. *British Medical Journal*. 2013. 03 Jun-09 Jun. f3591. doi: 10.1136/bmj.f3591 23744600

[4] Knights J, Salvatore M, Simpkins G, Hunter K, Khandelwal M. In search of best practice for postpartum depression screening: is once enough? *European Journal of Obstetrics*, *Gynecology*, *and Reproductive Biology*. 2016. 206:99–104. doi: 10.1016/j.ejogrb.2016.08.030 27664907

[5] American Pregnancy Association. *Depression during pregnancy*: *Signs*, *symptoms*, *and treatment*. 2021a. https://americanpregnancy.org/healthy-pregnancy/pregnancy-health-wellness/depression-during-pregnancy/#:~:text=Pregnancy%20is%20supposed%20to%20be%20one%20of%20the,struggle%20with%20some%20symptoms%20of%20depression%20during%20pregnancy.

[6] American Pregnancy Association. *How to prevent postpartum depression*. 2021b. https://americanpregnancy.org/healthy-pregnancy/first-year-of-life/how-to-prevent-postpartum-depression/

[7] Tyokighir D, Hervey A, Schunn C, Clifford D, Ahlers-Schmidt C. Qualitative assessment of access to perinatal mental health care: A social-ecological framework of barriers. *Kansas Journal of Medicine*, 2022. 15(1), 48–54. 10.17161/kjm.vol15.15853. 35371389 PMC8942588

[8] Yu M, Sampson M. Pediatrician attitudes and practices regarding postpartum depression screening: Training and interprofessional collaboration needed. *Journal of Interprofessional Education & Practice*, 2019. 15,1–4.

[9] Hsieh WJ, Sbrilli M, Huang WD, Hoang TM, Meline B, Laurent H. et al. Patients’ perceptions of perinatal depression screening: A qualitative study. *Health Affairs*, 2021. 40(10), 1612–1617. doi: 10.1377/hlthaff.2021.00804 34606357

[10] Tabb KM, Hsieh WJ, Sung JS, Hoang TMH, Deichen Hansen ME, Lux E, et al. Patient engagement to examine perceptions of perinatal depression screening with the capabilities, opportunities, motivation, and behaviors (COM-B) model. *Frontiers in health services*, 2022. 2, 845441. doi: 10.3389/frhs.2022.845441 36925830 PMC10012820

[11] U.S. Preventive Services Task Force: Curry S, Krist A, Owens D, Barry M, Caughey A, Davidson K, et al. Interventions to prevent perinatal depression: U.S. Preventive Services Task Force Recommendation Statement. *Journal of the American Medical Association (JAMA)*. 2019. 321(6):580–587. doi: 10.1001/jama.2019.0007 . https://pubmed.ncbi.nlm.nih.gov/30747971/30747971

[12] Loudon H, Nentin F, Silverman ME. Using clinical decision support as a means of implementing a universal postpartum depression screening program. *Archives of Women’s Mental Health*, 2016. 19, 501–505. doi: 10.1007/s00737-015-0596-y 26669601

[13] Viveiros C, Darling E. Barriers and facilitators of accessing perinatal mental health services: The perspectives of women receiving continuity of care midwifery, *Midwifery*, 2018. 65, 8–15. doi: 10.1016/j.midw.2018.06.018 30029084

[14] Agresti A. *Categorical data analysis*. 2002. 2^nd^ edition. John Wiley & Sons, Inc.

[15] R Core Team. *R*: *A language and environment for statistical computing*. R Foundation for Statistical Computing, Vienna, Austria. https://www.R-project.org/. 2023.

[16] Cox J, Holden J, Sagovsky R. Detection of postnatal depression. Development of the 10-item Edinburgh Postnatal Depression Scale. *British Journal of Psychiatry*. 1987. 150:782–6. doi: 10.1192/bjp.150.6.782 3651732

[17] Kroenke K, Spitzer RL, Williams J. The PHQ-9. *Journal of General Internal Medicine*. 2001. 16, 606–613. 11556941 10.1046/j.1525-1497.2001.016009606.x PMC1495268

[18] National Institutes of Mental Health (NIMH). *Perinatal depression*. 2023. https://www.nimh.nih.gov/health/publications/perinatal-depression

[19] Clevesy M, Gatlin T, Cheese C, Strebel K. A project to improve postpartum depression screening practices among providers in a community women’s healthcare clinic. *Nursing for Women’s Health* .2019. 23(1), 21–30. doi: 10.1016/j.nwh.2018.11.005 30605631

[20] Sidebottom A, Vacquier M, LaRusso E, Erickson D, Hardeman R. Perinatal depression screening practices in a large health system: Identifying current state and assessing opportunities to provide more equitable care. *Archives in Women’s Mental Health*. 2021. 24, 133–144 (2021). 10.1007/s00737-020-01035-x PMC7929950 32372299

[21] Branquinho M, Canavarro M, Fonseca A. A blended psychological intervention for postpartum depression: acceptability and preferences in women presenting depressive symptoms, *Journal of Reproductive and Infant Psychology*, 2023. 41 *(*1), 78–92, doi: 10.1080/02646838.2021.1969350 34420466

[22] Sufredini F, Catling C, Zugai J, Chang S. The effects of social support on depression and anxiety in the perinatal period: A mixed-methods systematic review. *Journal of Affective Disorders*, 2022. 319, 119–141, doi: 10.1016/j.jad.2022.09.005 36108877

[23] Carter R, Cust F, Boath E. Effectiveness of a peer-support intervention for antenatal depression: A feasibility study. *Journal of Reproductive and Infant Psychology*, 2020. 38(3), 259–270. doi: 10.1080/02646838.2019.1668547 31538488

[24] Waqas A, Koukab A, Meraj H, Dua T, Chowdhary N, Fatima B, et al. Screening programs for common maternal mental health disorders among perinatal women: Report of the systematic review of evidence. *BMC Psychiatry*. 2022. 22(1):54. doi: 10.1186/s12888-022-03694-9 ; PMCID: PMC8787899. 35073867 PMC8787899

[25] Earls M, Yogman M, Mattson G, Rafferty, J. Incorporating recognition and management of perinatal depression into pediatric practice. Committee on psychosocial aspects of child and family health. *Pediatrics*. 2019. 143(1), e20183259. 10.1542/peds.2018-3259 30559120

[26] American College of Obstetricians & Gynecologists (ACOG). *Implementing perinatal mental health screening*. 2024. https://www.acog.org/programs/perinatal-mental-health/implementing-perinatal-mental-health-screening

[27] McKean M, Caughey A, Yuracko McKean M, Cabana M, Flaherman V. Postpartum depression: When should healthcare providers identify those at risk? *Clinical Pediatrics*, 2018. 57(6), 689–693. doi: 10.1177/0009922817733696 28969467

[28] Docherty A, Najjar R, Combs S, Woolley R, Stoyles S. Postpartum depression screening in the first year: A cross-sectional provider analysis in Oregon. *Journal of the American Association of Nurse Practitioners*. 2020. 32(4). p 308–315. doi: 10.1097/JXX.0000000000000250 31373961

[29] National Association of Pediatric Nurse Practitioners. (NAPNAP). Position statement on the integration of mental health care in pediatric primary care settings. *Journal of Pediatric Health Care*. 2020. 34(5),514–517. https://www.jpedhc.org/article/S0891-5245(20)30128-0/pdf 10.1016/j.pedhc.2007.06.006 17990377

[30] Sorg M, Coddington J, Ahmed A, Richards E. Improving postpartum depression screening in pediatric primary care: A quality improvement project. *Journal of Pediatric Nursing*, 2019. 46, 83–88. doi: 10.1016/j.pedn.2019.03.001 30865875

[31] Hutchens B, Kearney J. Risk factors for postpartum depression: An umbrella review. *Journal of Midwifery &* *Women’s Health*, 2020. 65, 96–108. doi: 10.1111/jmwh.13067 31970924

[32] Bina R. Predictors of postpartum depression service use: A theory-informed, integrative systematic review. *Women and Birth*, 2020. 33, e24–e32. doi: 10.1016/j.wombi.2019.01.006 30718105

