# Correction: The role of maternal and child healthcare providers in identifying and supporting perinatal mental health disorders

**DOI:** 10.1371/journal.pone.0331148

**Published:** 2025-08-26

**Authors:** Carmen Kiraly, Betty Boyle-Duke, Liat Shklarski

There is an error in affiliation 3 for author Liat Shklarski. The correct affiliation 3 is: Silberman School of Social Work, Hunter College, New York, NY, USA.
