# Supplementary material for: The role of maternal and child healthcare providers in identifying and supporting perinatal mental health disorders
Source: PLoS One. 2024 Jul 11;19(7):e0306265. doi: 10.1371/journal.pone.0306265 (PMC11239045; doi:10.1371/journal.pone.0306265)
Supplement: S1 File — (DOCX) [file pone.0306265.s001.docx]

Obstetrician survey

[https://cunyhunter.co1.qualtrics.com/jfe/form/SV_7TDMCGYEnrYiSyN](https://urldefense.com/v3/__https:/cunyhunter.co1.qualtrics.com/jfe/form/SV_7TDMCGYEnrYiSyN__;!!DeIc-uvKXH9G!pWAHMG5NQ6dtUjlumA3mNktoXi8yLut6lkcw6r-r1_GctpNedMnNflTDl_E30gNRKj0$)​

Pediatrician/NP survey
[https://cunyhunter.co1.qualtrics.com/jfe/form/SV_78tIETxvPI5iqNf](https://urldefense.com/v3/__https:/cunyhunter.co1.qualtrics.com/jfe/form/SV_78tIETxvPI5iqNf__;!!DeIc-uvKXH9G!pWAHMG5NQ6dtUjlumA3mNktoXi8yLut6lkcw6r-r1_GctpNedMnNflTDl_E3OU0d7Nw$)
